# Effect of Exergame Training and Detraining on Lower-Body Strength, Agility, and Cardiorespiratory Fitness in Women with Fibromyalgia: Single-Blinded Randomized Controlled Trial

**DOI:** 10.3390/ijerph17010161

**Published:** 2019-12-24

**Authors:** Santos Villafaina, Yolanda Borrega-Mouquinho, Juan Pedro Fuentes-García, Daniel Collado-Mateo, Narcis Gusi

**Affiliations:** 1 Faculty of Sport Science, University of Extremadura, Avda: Universidad S/N, 10003 Cáceres, Spain; svillafaina@unex.es (S.V.); yborrega@alumnos.unex.es (Y.B.-M.); ngusi@unex.es (N.G.); 2 Centre for Sport Studies, Rey Juan Carlos University, 28943 Fuenlabrada, Madrid, Spain; danicolladom@gmail.com

**Keywords:** virtual reality, chronic pain, physical activity, chair–stand test, 10 step stair test, six-minute walk test

## Abstract

The aim of this study was to analyze the effects of a 24 week exergame intervention and 24 weeks of detraining on lower-limb strength, agility, and cardiorespiratory fitness in women with fibromyalgia (FM). It was performed as a single-blinded randomized controlled trial of 55 women with FM. University facilities were used. The 24 week exergame intervention was focused on mobility, postural control, upper- and lower-limb coordination, aerobic fitness, and strength. Participants performed 120 min of exergaming per week, which was divided into two sessions. Twenty-four weeks after the end of the intervention, participants were re-evaluated. A chair–stand test, 10 step stair test, and six-minute walk test were conducted to assess lower-body strength, agility, and cardiorespiratory fitness, respectively. The exergame intervention significantly improved lower-limb strength and cardiorespiratory fitness. However, no significant effects on agility were observed. After the detraining period, lower-limb strength and agility returned to their baseline level, but improvements in cardiorespiratory fitness were sustained over time. Exergaming was therefore shown to be beneficial for physical fitness in people with FM. However, exergames had to be played regularly to maintain the benefits. This long-term intervention (24 weeks) may have changed the lifestyle of women with FM, which could explain why cardiorespiratory fitness improvements remained after the detraining period. Future research should focus on lifestyle changes after long-term interventions.

## 1. Introduction

Fibromyalgia (FM) is a chronic syndrome characterized by widespread pain and other symptoms such as fatigue, sleep disorders, anxiety, depression, impaired balance, stiffness, risk of falling, poor physical condition, and cognitive dysfunction [1,2]. These symptoms lead to a reduction in the quality of life of women with FM [3].

There is currently no single treatment for patients with FM (either nonpharmacological or pharmacological). Revised guidelines from the European League Against Rheumatism (EULAR) suggested that treatment should be based on a nonpharmacological approach that involves exercise. This is because exercise is relatively cheap and plays a role in pain reduction [4,5]. Therefore, exercise has the largest body of supporting evidence among all nonpharmacological therapies for its role in the reduction of symptoms associated with FM [6].

Previous studies have shown that exercise interventions based on aerobic exercise or strength can improve pain, depression, physical function, disease impact, and quality of life in women with FM [6,7,8,9,10,11,12,13,14]. Furthermore, exergame interventions (a nonimmersive form of virtual reality) that involve physical exercise [15] have previously been used in patients with FM. Results indicated that exergames are useful to enhance mobility, quality of life, physical function, and brain dynamics in patients with FM [16,17,18,19,20].

However, improvements made during the intervention may be lost when the training regimen is interrupted. For example, after 14 days of detraining following an aerobic intervention in a healthy population, peak VO2 significantly decreased [21,22]. In patients with FM, previous studies have shown that most improvements derived from exercise interventions disappeared after the detraining period [23,24]. However, this finding is controversial because a previous study showed that benefits attained by regular exercise can be maintained for 30 months [25].

The present study aimed to evaluate the effects of a 24 week exergame-based intervention on lower-body strength, agility, and cardiorespiratory fitness in women with FM. It also aimed to observe the impact of a detraining period (24 weeks) after completing the exergame-based intervention. This is relevant because the effects of detraining after an exergame-based intervention have never been studied in patients with FM.

## 2. Materials and Methods

### 2.1. Trial Design

This study was a single-blinded randomized controlled trial. It was approved by the ethics committee of the University of Extremadura (62/2017). Participants gave their written consent for the procedures in the study. The trial was registered with the International Standard Randomized Controlled Trial Number Registry (ISRCTN65034180), and the protocol was published in https://doi.org/10.1186/ISRCTN65034180.

The current study focused on the effects of 24 weeks of exergame-based training and a 24-week detraining period in patients with FM. Three articles focusing on quality of life, physical fitness under dual-task conditions, and brain processing were recently published elsewhere [18,19,20]. The hypothesis of the current study was entirely different from previous studies, and included follow-up evaluation. This study also involved other specific research professionals and targeted a different audience.

### 2.2. Participants

A total of 56 participants were recruited from a local FM association (until 31 December 2017) and fulfilled the following inclusion criteria:

- Female and aged between 30 and 75 years;
- able to communicate with research staff;
- had given their informed consent; and
- diagnosed with FM by a rheumatologist according to 2010 American College of Rheumatology criteria [1].

Furthermore, participants were excluded if they (a) had changed their usual care therapies during the 24 weeks of the treatment, (b) had had contraindications for physical-exercise programs, or (c) were pregnant. 

Random numbers were assigned to participants, and then they were randomly allocated in a group (control or exergame) by an investigator. This investigator did not participate in data acquisition or statistical analyses. All evaluations were performed by another investigator who was blinded to the grouping allocation. Participants were unable to be blinded given they were informed of the procedures.

### 2.3. Intervention

Participants that were enrolled in the exergame group conducted a 24 week exercise intervention with 2 sessions per week, each of 1 h duration. The intervention program consisted of an exergame (VirtualEx-FM) designed by the research group to improve the fitness and ability of women with FM to assist in activities of daily living. This exergame was previously used, and its main characteristics are published elsewhere [16].

The objective of VirtualEX-FM was to increase the attendance and motivation of FM patients while taking into account the preferences [26] and needs [27] of participants. The exergame fulfilled the 8 key criteria for consideration as an adequate virtual-reality rehabilitation tool [26]. VirtualEx-FM focused on postural control, coordination of the upper and lower limbs, aerobic fitness, strength, and mobility, while taking into account quality of movements. Furthermore, the intervention was supervised and controlled by a technician [16].

A typical session involved the following:

- A warm-up where participants were guided by a video made by a kinesiologist;
- aerobic exercises based on dance steps shown by a dance teacher;
- postural control and coordination games where participants had to reach for an apple that came and went in different locations around them (the kinesiologist could manually control the body part that participants had to use to reach the apple); and
- walking training where participants had to follow a virtual trail of footprints. The type and amplitude of steps were controlled.

The control group continued with their usual daily life. This included remaining on medications.

### 2.4. Outcomes

The primary outcomes of the present study were lower-limb strength, agility, and cardiorespiratory fitness as measured by the chair–stand test, 10 step stair test, and 6 min walk test, respectively. Data were collected in the Faculty of Sport Science (Cáceres, Spain) facilities. 

The chair–stand test evaluated lower-limb strength by repeating the action of standing up from a sitting position (i.e., until reaching complete knee extension) and then sitting down as many times as possible in 30 s [28]. A previous study of women with FM [29,30] reported excellent reliability for this test with an intraclass correlation coefficient (ICC) and confidence interval of 0.91 (0.87–0.94).

The 10 step stair test evaluated agility by measuring the time it took to climb 10 steps. Participants had to climb ‘as quickly and as safely as possible’. The use of handrails when necessary was permitted for safety reasons. This test showed excellent reliability (ICC = 0.972) in a previous study in women with FM [31].

The 6 minute walk test was used to measure cardiorespiratory fitness. It determined the maximum distance (in meters) that patients could walk in 6 min following a rectangular path of 45.7 m [28,32]. A previous study [29] reported the excellent reliability of this test in women with FM, with an ICC and confidence interval of 0.92 (0.87–0.94).

A revised version of the Fibromyalgia Impact Questionnaire (FIQ-r) was selected to evaluate the impact of the disease [33]. The total score ranged from 0 to 100, calculated as the sum of 3 dimensions: function domain (9 items and score 0–30), symptom domain (10 items and score 0–50), and overall impact (2 items and score 0–20). The Spanish version of the FIQ-r was used in the present study [34]. 

The International Physical Activity Questionnaire (IPAQ) was used to monitor physical activity and inactivity. It was employed to calculate total Metabolic Equivalents (METs) per week and sitting time (minutes per day) [35].

Total METs per week was calculated according to instructions from the IPAQ research committee [36] as follows:

Total Work MET-minutes/week = sum of Walking + Moderate + Vigorous MET-minutes/week. 

In this regard, the walking, moderate, and vigorous MET-minutes/week were calculated as:

- Walking MET-minutes/week = 3.3 × walking minutes × walking days;
- moderate MET-minutes/week = 4.0 × moderate-intensity activity minutes × moderate-intensity days; and
- vigorous MET-minutes/week = 8.0 × vigorous-intensity activity minutes × vigorous-intensity days.

These tests and questionnaires were performed before and after the exergame intervention (24 weeks), as well as after 24 weeks of detraining.

### 2.5. Statistical Analysis

Sample size was calculated according to data extracted from the FIQ-r [37]. A 14% reduction in the total FIQ-r score was considered clinically important [38]. It was expected that the average FIQ-r would be 70.5 (11.8) on the basis of data from a previous study of Spanish women with FM [39]. The minimum power required to detect at least 14% difference between groups was found to be 85% with an α-value of 0.05. Therefore, a minimum of 26 participants per group was required.

Parametric tests were conducted on the basis of results from the Shapiro–Wilk and Kolmogorov–Smirnov tests. Independent-sample *t*-tests were conducted to explore baseline differences.

In order to evaluate the effects of the exergame intervention, repeated-measures ANOVA was performed. The model included the 2 groups (exergame and control) and the 3 different time points (before, after, and follow-up) at which outcomes were evaluated (2 × 3 ANOVA). Subsequently, we conducted intergroup and within-group analyses to characterize differences. 

To observe the evolution of each group (exergame and control) between different time points (before, after, and follow-up), *t*-tests for related samples were performed. Furthermore, *t*-tests for independent samples were conducted to detect intergroup differences at different time points. The alpha-level of significance (set at 0.05) was adjusted by the Benjamini–Hochberg procedure in order to control for false-discovery rates [40]. In order to represent intergroup and within-group results, GraphPad Prism software (version 6) (GP Prism-Inc, San Diego, CA, USA) was used to create a figure for each physical-fitness test.

The effect size (partial eta squared) was reported for each statistical test [41]. In accordance with Cohen [42], effect sizes were classified as small (0.01 ≤ η^2^ < 0.06), medium (0.06 ≤ η^2^ < 0.14), or large (η^2^ ≥ 0.14). A listwise deletion method was applied for intention-to-treat analysis. Statistical analyses were performed using statistical package Statistical Package for Social Sciences (SPSS 24.0; IBM Corp., Armonk, NY, USA).

## 3. Results

**Figure 1:**
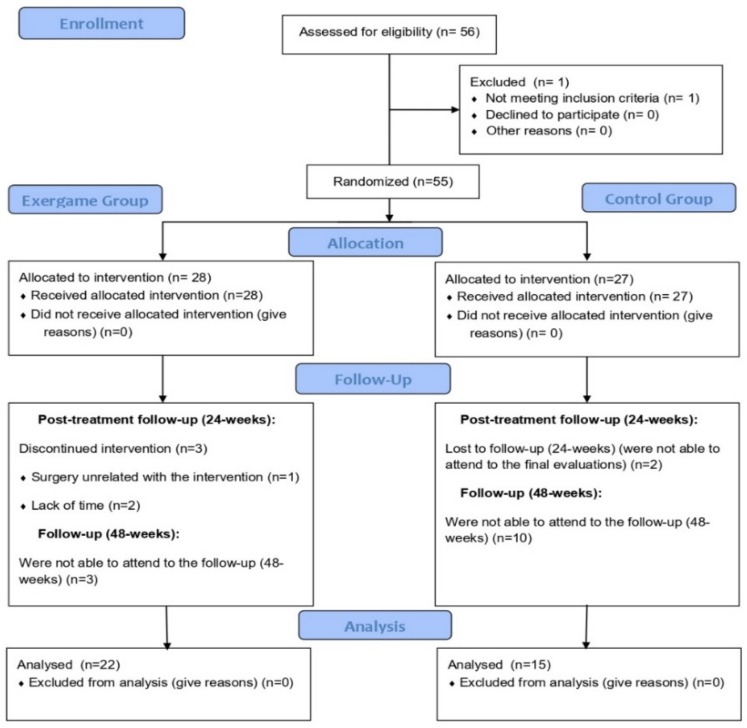
Flow chart of participants.

Figure 1 shows the flow diagram of participants according to the Consort 2010 (the checklist is provided as Supplementary Materials, Table S1). A total of 56 women with FM participated in the study. After being randomly divided into two groups, 50 women completed the intervention (25 in the exergame group and 25 in the control group). However, six months after the end of the intervention (48 weeks follow-up), only 22 participants from the exergame group and 15 participants from the control group attended the evaluation. Importantly, side effects of the exergame intervention were not detected.

Table 1 shows values for different physical fitness tests, total FIQ-r score, age, METs per week, sitting time, body-mass index, and fat mass at baseline. Differences were not found in any of these variables.

Repeated-measures ANOVA showed significant effects on the chair–stand test (*p*-value = 0.017) and six-minute walk test (*p*-value = 0.011; Table 2). However, no significant effect was found for the 10 step stair test (*p*-value = 0.666; Table 2). Differences in the chair–stand test and six-minute walk test were large, as shown by effect-size values.

Independent-sample *t*-tests indicated that the exergame intervention significantly improved performance in the chair–stand test (*p*-value = 0.003) and the six-minute walk test (*p*-value = 0.003). However, no significant differences were found in the 10 step stair test (*p*-value = 0.392).

**Figure 2:**
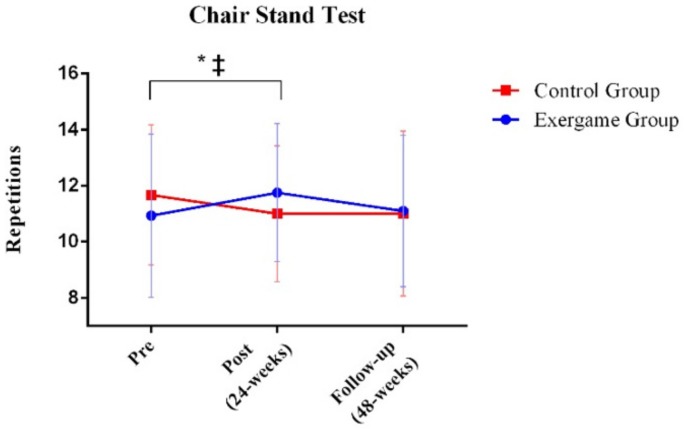
Comparison between exergame and control groups in the three time points for the Chair Stand Test. * The exergame group significantly increased the number of repetitions (*p*-value <0.05); ‡ The control group significantly decreased the number of repetitions (*p*-value <0.05).

**Figure 3:**
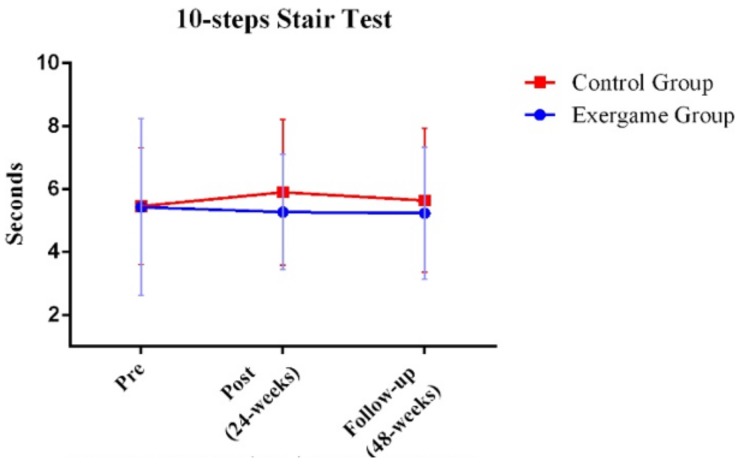
Comparison between exergame and control groups in the three time points for the 10-steps Stair Test. Significant effects were not found (*p*-value > 0.05).

**Figure 4:**
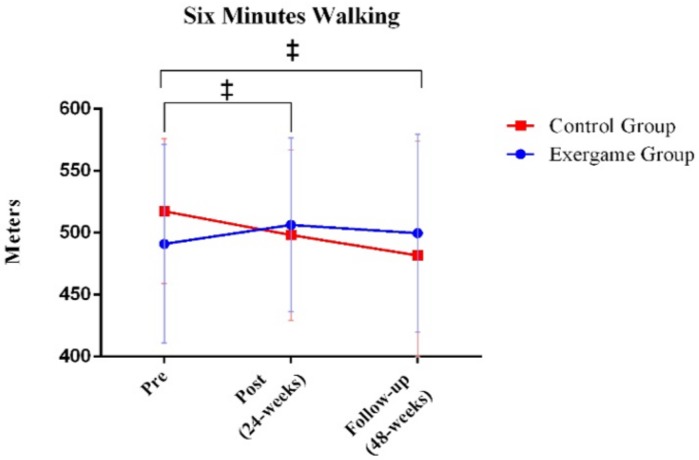
Comparison between exergame and control groups in the three time points for the 6-min Walking Test. ‡ The control group significantly decreased the number of meters (*p*-value < 0.05).

In order to assess within-group differences at different time points (before, after, and follow-up), paired-sample *t*-tests were conducted. For the chair–stand test, performance in the exergame group significantly increased after intervention (*p*-value = 0.030) while performance significantly decreased in the control group (*p*-value = 0.046; Figure 2). Differences were not observed at any time point in the 10 step stair test (Figure 3). The exergame group did not significantly improve in the six-minute walk test. However, the performance of the control group significantly decreased in this test when comparing the pre- and postintervention evaluations (*p*-value = 0.015) and follow-up with the preintervention evaluation (*p*-value = 0.015; Figure 4). 

Six months after completing the exergame intervention (48 weeks), follow-up evaluation was performed. Independent-sample *t*-tests indicated that lower-body strength and agility returned to baseline after six months of detraining (*p*-value > 0.05), whereas cardiorespiratory fitness was maintained over time (*p*-value = 0.013).

Paired-sample *t*-tests and *t*-tests of independent samples did not show any significant changes in total FIQ-r score, METs per week, or sitting time (*p*-value > 0.05).

## 4. Discussion

The main objective of this study was to evaluate the effects of exergame intervention on lower-body strength, agility, and cardiorespiratory fitness. It also aimed to observe the impact of six months of detraining in women with FM. Results indicated that the exergame intervention improved lower-body strength and cardiorespiratory fitness. Interestingly, cardiorespiratory fitness was maintained over time, while improvements in lower-body strength returned to baseline levels after six months of detraining.

Previous studies that focused on the detraining period in women with FM showed that improvements returned to baseline levels after this period [23,24]. This was somewhat similar to our results, where lower-body strength returned to baseline levels after six months of detraining. However, cardiorespiratory fitness improvements mostly remained after the detraining period. An explanation for this could be that participants changed their lifestyle after long-term intervention (six months). This may result in women with FM being more active even after the end of the exergame intervention.

When analyzing IPAQ data, we observed that the control-group participants were less active than exergame participants were after the detraining period. On average, control-group participants were sedentary for around 45 min more per day than exergame participants were (Table 2). This could indicate that they continued to perform physical activity even after the intervention had finished. This is relevant because the replacement of 30 min of sedentary time with physical activity leads to improvement in health-related quality of life, FM impact [43], and quality of sleep [44]. Future studies should investigate the effects of long-term exergame interventions on the lifestyle of women with FM.

FM has significant impact on activities of daily living due to the associated chronic pain and fatigue [1,2] that reduce quality of life [3]. Exergames are considered a useful tool to improve the adherence, frequency, and duration of exercise interventions [45,46]. This, in turn, leads to improved mobility, balance, pain, and fear of falling [17,47]. In the present study, improvements in lower-body strength and cardiorespiratory fitness were observed in those undertaking the exergame intervention. No improvements in agility (as measured by the 10 steps stair test) were observed. This finding agrees with a previous study that reported improvements in physical fitness in a 24-week exergame intervention. However, the effects of the intervention were limited to upper-limb strength and mobility under dual-task conditions [19].

Aside from fitness parameters, previous studies of exergames in women with FM reported improvements in health-related quality of life, disease impact, pain, and brain processing [16,17,18,19,20]. The 24 week exergame intervention in this study did not have any significant effect on disease impact. A potential explanation for this nonsignificant effect is the relatively low disease impact in women included in the study, with mean FIQ-r = 53.17 (16.93) at baseline, 51.60 (18.25) after intervention, and 51.10 (18.99) after the 48 week follow-up. This may have led to a floor effect. The mean (SD) in the validation study of the Spanish version of the FIQ-r was 68.2 (17.5) [34], and the cut-off point for severe FM symptoms was set at 60. Evidently, our participants had moderate disease impact, and it is possible that intervention effects were underestimated due to a floor effect. However, a shorter intervention (eight-week exergame intervention with the same protocols and involving women with moderate FM symptoms according to the FIQ score) showed a significant effect on disease impact. Therefore, future studies should explore the influence of protocol duration on disease impact in women with FM. Another potential explanation for the results is the novelty of the exercise protocol. Motivation to play exergames might be reduced when the duration of the protocol is increased, which may be related to the lack of different exercises and alternatives. Therefore, future studies with duration longer than six months may consider adjusting not only intensity and repetitions, but also the types of exercises. Furthermore, measures of motivation and adherence should be included to test this hypothesis.

Adherence to physical exercise is a well-documented issue in women with FM [48]. In our intervention, 89% of participants completed the 24 week intervention. This completion rate is higher than in aerobic interventions in which the average drop-out rate is around 22% [49]. However, in a previous exergame intervention, adherence was even higher than that in our study (98%). This could be due to the duration of the intervention, which was significantly shorter than that in our study (eight weeks). Moreover, in the present study, in order to promote adherence, the exergame intervention was supervised by a physical therapist, as suggested by Lewis and Rosie [26]. Classes were also performed in groups (two or three participants per group) [50]. Future studies should incorporate these strategies in order to improve adherence to physical-exercise interventions in women with FM.

This study had some limitations. First, there were a significant number of women lost by the 48 week follow-up (*n* = 18). Second, the study focused on women with FM, so results cannot be extrapolated to men with FM. Third, in order to monitor physical activity and inactivity, the IPAQ questionnaire was used. This questionnaire is not an objective measure, and future studies should consider using other instruments, such as multisensory armband accelerometers [51]. Fourth, considering reference values of the FIQ-r, it is possible that a floor effect was present in the assessment of FM impact. Effects of the intervention may therefore have been underestimated.

## 5. Conclusions

Exergaming improved lower-body strength and cardiorespiratory fitness in women with FM. After a 24 week detraining period, lower-body-strength improvements had disappeared, while improvements in cardiorespiratory fitness remained. Exergames must be performed regularly to maintain strength benefits. However, the length of intervention (24 weeks) may have changed the lifestyle of women with FM, which could explain why cardiorespiratory fitness improvements remained after the detraining period.

## Figures and Tables

**Table 1:** Demographic data and differences between groups at baseline.

	Exergame Group Mean (SD)	Control Group Mean (SD)	*p*-Value	Effect Size
Sample size (N)	22	15		
Age (years)	54.27 (9.29)	53.44 (9.47)	0.788	0.002
FIQ-r	52.62 (17.12)	54.97 (20.34)	0.702	0.004
METs (per week)	2667.3 (3704)	4422.71(4291)	0.174	−0.046
Sitting time (min per day)	264.09 (171.94)	276 (166.34)	0.836	<0.001
BMI (kg/m^2^)	27.11 (2.90)	28.19 (3.88)	0.332	0.026
Fat mass (kg)	26.99 (5.95)	26.9 (7.60)	0.969	<0.001
Chair–stand test (Rep)	10.93 (2.91)	11.31 (2.80)	0.688	0.005
Stairs (s)	5.43 (2.81)	5.46 (1.86)	0.980	<0.001
Six-minute walk test (M)	491.15 (80.21)	517.45 (58.42)	0.285	0.032

Note: FIQ-r, Fibromyalgia Impact Questionnaire; SD, standard deviation; BMI, body-mass index; kg, kilograms; MET, metabolic equivalent; Rep, repeats; s, seconds; m, meters.

**Table 2:** Effects of an exergame intervention in women with fibromyalgia on lower-body strength, agility, and cardiorespiratory fitness.

Test	Groups				Comparisons Between Groups		
		Preintervention Mean (SD)	Post-Treatment (24 Weeks) Mean (SD)	Follow-Up (48 Weeks) Mean (SD)	F	*p*-Value *	Effect Size
**FIQ-r**	Exergames (N = 22)	53.17 (16.93)	51.60 (18.25)	51.10 (18.99)	0.295	0.746	0.016
	Control (N = 15)	54.15 (19.98)	55.35 (20.11)	52.07 (18.37)			
**METs (per week)**	Exergames (N = 22)	2667.35 (3704.18)	2990.17 (3090.58)	3194.14 (3356.59)	0.624	0.541	0.034
	Control (N = 15)	4422.71 (4291.30)	3406.94 (6315.86)	3063.29 (2942.56)			
**Sitting time (min/day)**	Exergames (N = 22)	262.38 (178.04)	277.14 (162.36)	364.28 (205.07)	0.369	0.694	0.023
	Control (N = 15)	270 (171.92)	265.71 (129.89)	409.28 (205.71)			
**Chair–stand test (Rep)**	Exergames (N = 22)	10.93 (2.91)	11.75 (2.46)	11.10 (2.70)	4.593	0.017	0.213
	Control (N = 15)	11.67 (2.50)	11.00 (2.42)	11.00 (2.94)			
**Stairs (s)**	Exergames (N = 22)	5.43 (2.81)	5.27 (1.83)	5.23 (2.10)	0.412	0.666	0.024
	Control (N = 14)	5.46 (1.86)	5.90 (2.32)	5.64 (2.29)			
**Six-minute walk test (m)**	Exergames (N = 22)	491.15 (80.21)	506.47 (70.15)	499.74 (80.05)	5.191	0.011	0.234
	Control (N = 15)	517.45 (58.42)	498.21 (68.80)	481.70 (92.30)			

* *p*-value obtained from repeated-measures ANOVA. Note: F, Fisher´s F; FIQ-r, Revised version of the Fibromyalgia impact questionnaire; MET, Metabolic Equivalent; SD, standard deviation; Rep, repetitions; s, seconds; m, meters.

## Supplementary Materials

The following are available online at https://www.mdpi.com/1660-4601/17/1/161/s1, Table S1: CONSORT 2010 checklist of information to include when reporting a randomised trial.
